# Genomic structure analysis of a set of *Oryza nivara* introgression lines and identification of yield-associated QTLs using whole-genome resequencing

**DOI:** 10.1038/srep27425

**Published:** 2016-06-02

**Authors:** Xin Ma, Yongcai Fu, Xinhui Zhao, Liyun Jiang, Zuofeng Zhu, Ping Gu, Wenying Xu, Zhen Su, Chuanqing Sun, Lubin Tan

**Affiliations:** 1 National Center for Evaluation of Agricultural Wild Plants (Rice), Beijing Key Laboratory of Crop Genetic Improvement, Laboratory of Crop Heterosis and Utilization, MOE, Department of Plant Genetics and Breeding, China Agricultural University, Beijing 100193, China; 2 State Key Laboratory of Plant Physiology and Biochemistry, China Agricultural University, Beijing 100193, China

## Abstract

*Oryza nivara*, an annual wild AA-genome species of rice, is an important gene pool for broadening the genetic diversity of cultivated rice (*O. sativa* L.). Towards identifying and utilizing favourable alleles from *O. nivara*, we developed a set of introgression lines (ILs) by introducing *O. nivara* segments into the elite *indica* rice variety 93-11 background through advanced backcrossing and repeated selfing. Using whole-genome resequencing, a high-density genetic map containing 1,070 bin-markers was constructed for the 131 ILs, with an average length of 349 kb per bin. The 131 ILs cover 95% of *O. nivara* genome, providing a relatively complete genomic library for introgressing *O. nivara* alleles for trait improvement. Using this high-density bin-map, QTL mapping for 13 yield-related traits was performed and a total of 65 QTLs were detected across two environments. At ~36.9% of detected QTLs, the alleles from *O. nivara* conferred improving effects on yield-associated traits. Six cloned genes, *Sh4*/*SHA1*, *Bh4*, *Sd1*, *TE*/*TAD1*, *GS3* and *FZP*, colocalised in the peak intervals of 9 QTLs. In conclusion, we developed new genetic materials for exploration and use of beneficial alleles from wild rice and provided a basis for future fine mapping and cloning of the favourable *O. nivara*-derived QTLs.

Cultivated rice (*Oryza sativa* L.) is the staple food for billions of people worldwide and its production is essential for global food security. Utilization of heterosis and agronomically important genes has led to great progress in improving rice production during the past decades^1^,^2^. However, the sustainable increase of rice yield faces the challenges of global population explosion, rapid climatic change and shortages of water and land^3^,^4^. A key limiting factor for the long-term and sustained improvement of modern rice cultivars is genetic diversity, which has markedly decreased by strong artificial selection^5^,^6^. Discovery and deployment of favourable alleles from wild rice has been proposed as a key strategy for broadening the genetic diversity of cultivated rice and bringing a new green revolution in rice breeding.

Thousands of wild relatives of rice have been collected and are stored in germplasm banks. These germplasms offer a wide range of genetic resources for creating superior rice varieties in the future. However, it is difficult to utilize wild rice directly because of its overall inferior performance. The advanced backcross method is an effective strategy for identifying and introducing agronomically beneficial alleles from unadapted germplasm into the cultivated gene pool^7^. Following this strategy, many favourable alleles for yield-associated traits from *O. rufipogon*, a perennial wild rice, have been successfully identified. For example, some advanced backcross populations using the same *O. rufipogon* accession (IRGC 105491) as the donor parent and different *O. sativa* cultivar as the recurrent parent were developed and several valuable QTLs, of which alleles from the wild rice enhanced yield and yield components, were identified^8^,^9^,^10^,^11^,^12^. Additional researches have further confirmed that some QTLs truly improved the yield performance of elite cultivar or conferred beneficial effect to enhance the trait value of the breeding lines^12^,^13^. Many *O. rufipogon* introgression lines (ILs) have been developed by repeated selfing of advanced backcross populations and provide ideal genetic materials for evaluating agronomic traits and for positional cloning of interesting genes or QTLs, because of the simple and stable genetic background of wild rice ILs^14^,^15^,^16^,^17^,^18^. The annual diploid species *O. nivara* is another close wild relative of cultivated rice with great potential to improve elite cultivars, for instance, enhanced grassy stunt virus resistance and yield components has been achieved through transferring useful genes from *O. nivara* into cultivated rice varieties^19^,^20^,^21^. However, only few introgression libraries have been developed with *O. nivara*.

The rapid development of next-generation sequencing (NGS) technology has opened new opportunities for dissecting complex traits^22^,^23^. Compared with QTL mapping using conventional molecular markers, the whole-genome sequencing approach has more advantages, including the construction of a high-density genetic map based on high quality SNP markers, reduction of the amount of time and effort required for QTL mapping, and improving the accuracy and precision of QTL mapping for the complex traits. Using a whole-genome resequencing strategy, Huang *et al.*^24^ constructed a high-resolution bin map that delimited the largest effect QTL for plant height (PH) into a 100 kb physical interval. Wang *et al.*^25^ identified 49 loci associated with 14 agronomic traits using this map, of which 5 large-effect loci were mapped to relatively small chromosomal regions containing 5 strong candidate genes. Owing to its high resolution, accuracy and low cost, this strategy has also been widely applied in other crop plants, such as maize^26^,^27^, sorghum^28^ and soybean^29^. The combination of NGS and ILs provides a new strategy for exploiting the untapped reserve of wild alleles associated with agronomic traits.

In the present study, an IL population was constructed with the elite *indica* variety 93-11 as the recipient and *O. nivara* accession W2014 as the donor. Both parents and ILs were whole-genome resequenced to characterise the genomic structure of each IL. Using the high-density genetic map incorporating 1,070 bin-markers, a total of 65 QTLs for 13 yield-associated traits were detected, with favourable alleles from W2014 at 24 of these QTLs. Our finding indicated that the new set of ILs offers a unique opportunity for exploring and utilizing beneficial alleles from wild rice *O. nivara*.

## Results

### Genomic structure analysis of *O. nivara* ILs

#### Identification of SNPs of parents and 131 ILs

With respect to the reference genome (Os-Nipponbare-Reference-IRGSP-1.0, MSU release 7), the parents 93-11 and W2014 were resequenced with an estimated sequence depth of 13.55X and 13.64X, respectively, and the ILs with a mean depth of 2.83X. Using SAMtools software^30^, a total of 181,957 high-quality SNPs were identified between the two parents, with an average of 4.9 SNPs per 10 kb (Supplementary Dataset 1 and Supplementary Figure 1). Of these SNPs, 92,127 were located in the genic regions (Supplementary Table 1). Subsequently, we performed variant effect prediction analysis using SnpEff software^31^, and found that a total of 997 SNPs were predicted with high impact effect for protein sequences, either by altering the start codon, splice sites, or stop codon (Supplementary Dataset 2).

#### Construction of the bin map

**Figure 1:**
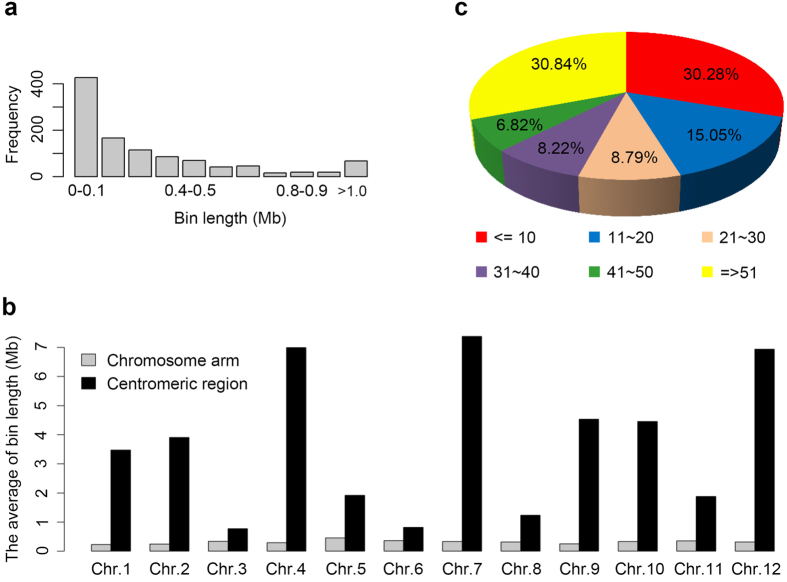
Characterization of bin markers. (**a**) Length distribution of 1,070 bins. (**b**) Comparison of bin length on chromosome arms and in centromeric regions. (**c**) Proportions of the bins containing different numbers of genes.

Following a previously reported procedure^24^, the genome-wide graphical genotypes of 131 ILs were identified. Based on the analysis of recombination breakpoints, a high-density bin map consisting of 1,070 bin markers was constructed (Supplementary Dataset 3). The average physical length of a bin was 349 kb, ranging from 5.0 kb to 7.37 Mb (Fig. 1a). The bins near centromeric regions were generally with larger length than those in the distal regions of chromosomes, which is expected because of the varied recombination rates in chromosome (Fig. 1b). On an average, each bin harboured 53 genes, and 324 (30.28%) bins contained fewer than 10 genes (Fig. 1c), indicating that the resolution of a bin map is much higher than that of a conventional map made using PCR-based markers. In the IL population, a total of 1,164 crossover events (COs) were detected with an average of 8.89 per IL (Supplementary Figure 2). There was a linear correlation between the length of chromosomes and the average number of COs per chromosome (Supplementary Figure 2).

#### Number, length and distribution of the introgressed segments

**Figure 2:**
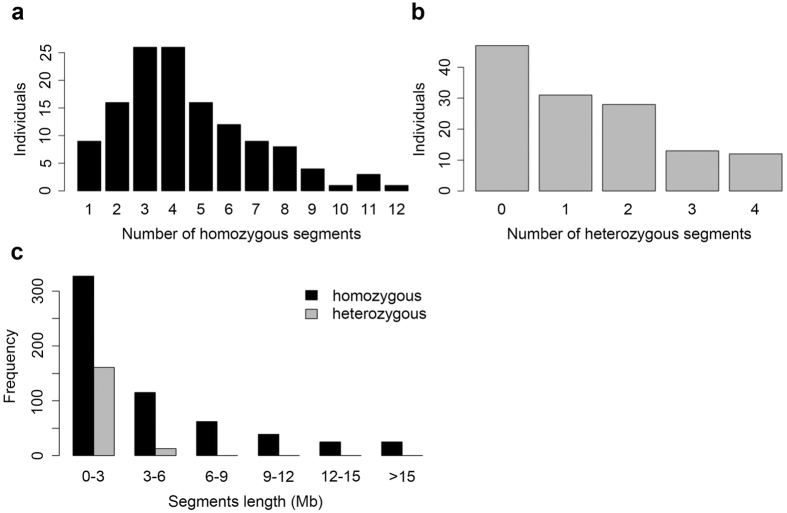
Frequency distribution of numbers of homozygous (**a**) and heterozygous (**b**) introgressed segments identified in ILs. (**c**) Length distribution of introgressed segments.

**Figure 3:**
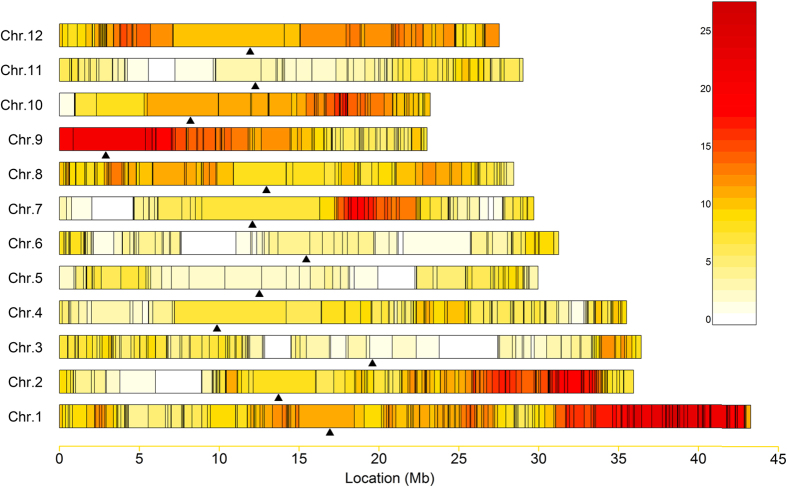
Coverage ratio of introgressed segments at the whole-genome level. Physical positions are based on the reference genome Nipponbare (Os-Nipponbare-Reference-IRGSP-1.0, MSU release 7). Black lines on chromosomes represent the recombination breakpoints. Centromeres are indicated by black arrows. The gradual change of colours on the bars indicates different numbers of introgressed segments.

The 131 ILs carried a total of 767 chromosomal segments from *O. nivara* in the genetic background of 93-11, including 593 homozygous and 174 heterozygous segments (Supplementary Table 2). On an average, each IL carried 5.8 introgressed segments with from 1 to 12 for homozygous segment and from 0 to 4 for heterozygous (Fig. 2, Supplementary Table 2). Forty-seven lines carried only homozygous segments. Additionally, we observed an uneven distribution of introgressed segments among the 12 chromosomes (Fig. 3). The long arms of chromosome 1 (physical interval: 32–42 Mb), chromosome 2 (26–33 Mb), and the short arm of chromosome 9 (0–10 Mb) carried more W2014 segments than other chromosomal regions.

**Figure 4:**
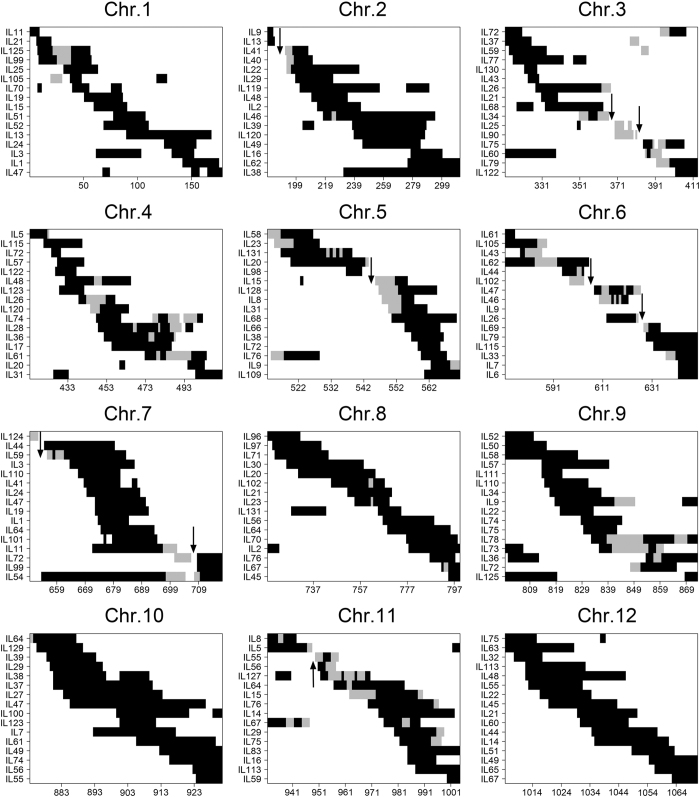
Introgression of chromosomal segments from an annual wild rice accession W2014 (*O*. *nivara*) along 12 chromosomes. The black regions represent homozygous chromosomal introgressed segments from wild rice W2014. The grey regions indicate heterozygous chromosomal regions (W2014/93-11). The white background indicates 93-11 homozygous genotype. Black arrows indicate gap regions that were not covered by introgressed segments. The x-axis represents the number of bins and the y-axis indicates the ILs that carried the introgressed segments.

The total size of the introgressed segments detected in the 131 ILs was 2.83 Gb, 7.57 times the total length of the rice genome. The average length of the *O. nivara*-derived segments carried by each IL was 21.73 Mb, 0.058 times the length of the genome. The average length of single introgressed segment was 3.7 Mb, ranging from 0.17 to 24.7 Mb. Of the introgressed segments, 76.4% were shorter than 5.0 Mb and 9.65% were longer than 10.0 Mb (Fig. 2c). The average length of the homozygous introgressed segments carried by each IL was 4.3 Mb and that of the heterozygous segments was 1.14 Mb (Supplementary Table 2). The coverage of the *O*. *nivara* genome by the ILs was 94.96% and the coverage of introgressed segments per chromosome varied from 85.40% on chromosome 3 to 100.00% on chromosomes 1, 4, 8, 9, 10, and 12 (Fig. 4 and Supplementary Table 2).

### Identification of yield-associated QTLs using *O. nivara* ILs

#### Trait segregation and field performance

To detect QTLs for yield-associated traits, we evaluated these ILs for 13 agronomic traits at two sites, Beijing and Hainan. Twelve agronomic traits showed normal distributions, while plant height (PH) showed bimodal distribution (Supplementary Figure 3). Significant correlations (*P* < 0.05) were observed among many traits at both sites. YPP at both sites showed strong positive correlations with PPL, PL, SBN, SNSB and SPP. At both sites, significantly negative correlations were observed between PPL and PL, between PPL and TGW and between PL and GW (Supplementary Tables 3 and 4).

#### Evaluation of the quality and accuracy of the high-density bin map

To evaluate the quality and accuracy of the high-density bin map of the ILs, we investigated 2 high heritability traits, Seed shattering (SH) and hull colour (HC), and performed QTL analysis. A major QTL, *qSH4*, controlling SH, was mapped to Bin 503 on the long arm of chromosome 4 in both environments, explaining 72.1% and 86.8% of the phenotypic variation, respectively. Bin 503 contains the *sh4*/*SHA1* gene, a transcription factor underlying the transition from shattering to non-shattering during rice domestication^32^,^33^ (Supplementary Figure 4).

Another major QTL for HC, *qHC4*, was detected in Bin 456 on chromosome 4 in both environments, explaining 55.1% and 90.9% of the phenotypic variation, respectively. The *Bh4* gene, conferring the change from black to yellow hull colour during rice domestication^34^, was located at ~227 kb in Bin 456 (Supplementary Figure 4). Taken together, these results strongly indicate that the high-density bin map of *O*. *nivara* ILs constructed in this study is robust for yield-associated QTL analysis.

#### QTL identification

**Figure 5:**
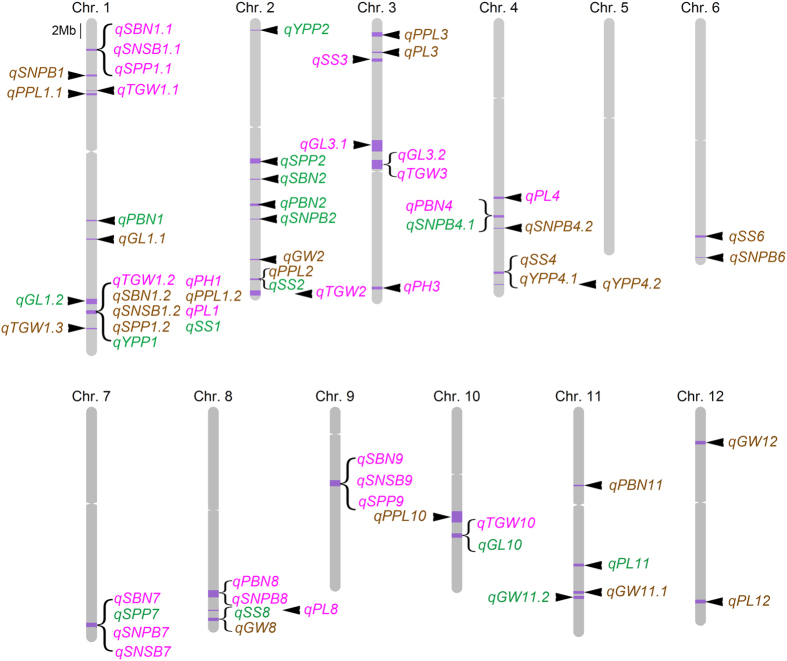
Locations of yield-associated QTLs on 12 chromosomes. The physical locations are based on the rice reference genome (Os-Nipponbare-Reference-IRGSP-1.0, MSU release 7). Green, brown and pink patterns represent QTLs detected in Beijing, Hainan and both sites, respectively. Brackets in bold indicate QTL hot spots.

A total of 65 QTLs were detected above an empirically determined experiment-wise significance threshold. Twenty-one (28.4%) significant QTLs were identified at both sites. The phenotypic variation explained by individual QTLs varied from 6.9% to 92.0%, and 51 QTLs explained more than 10% variation (Table 1 and Fig. 5). The *O*. *nivara* allele conferred positive effects at ~36.9% of the yield and yield component QTLs.

#### Plant height

Two QTLs for plant height were detected along chromosomes 1 and 3 at two sites, respectively. Phenotypic variation was explained by individual QTL ranging from 8.8% to 92.0%. All loci from *O. nivara* showed consistent positive effects that increase plant height.

#### Panicle number per plant

Five QTLs for panicle number per plant were detected, located on chromosomes 1, 2, 3 and 10. At 4 QTLs, the *O. nivara* alleles increased panicle number per plant. The *O. nivara*-derived allele at *qPPL1.2*, detected in Bin 150 on chromosome 1 in Hainan, with a maximum R^2^ of 29.9%, reduced panicle number in the *indica* variety 93-11 background.

#### Panicle length

Six QTLs for PL were identified. The *O. nivara* alleles at *qPL3* and *qPL4* deceased PL, whereas the *O. nivara* alleles at the other 4 QTLs increased PL. The 3 *O. nivara*-derived QTLs *qPL1*, *qPL4*, and *qPL8* were detected at both sites.

#### Primary branch number

Five QTLs were significantly associated with PBN. The phenotypic variation explained by these individual QTL ranged from 7.8% to 26.6%. Two QTLs, *qPBN4* and *qPBN8*, were identified at both sites. *qPBN4* explained 9.6% and 8.3% of the phenotypic variation at the two sites, respectively, with the allele from wild rice reducing branching. *qPBN8* explained 16.1% and 26.6% of the phenotypic variation at the two sites and the *O. nivara* allele increased primary panicle branches.

#### Spikelet number on primary branch

Seven QTLs for SNPB were detected on chromosomes 1, 2, 4, 6, 7 and 8. The phenotypic variation ranged from 7.2% to 28.1%. Two QTLs, *qSNPB7* and *qSNPB8*, were identified at both sites, and the *O. nivara* allele at both QTLs increased the spikelet number on the primary branch, whereas the *O. nivara* allele at the other 4 QTLs decreased the spikelet number on the primary branch in the 93-11 background.

#### Secondary branch number

Five QTLs were significantly associated with secondary branch number, and the phenotypic variation explained by individual QTLs varied from 7.6% to 18.4%. The *O. nivara* allele at *qSBN1.2* increased SBN, whereas the *O. nivara* alleles at the other 4 QTLs reduced it.

#### Spikelet number on secondary branch

Four QTLs for spikelet number on secondary branch were mapped on chromosomes 1, 7 and 9. The phenotypic variation explained by individual QTLs ranged from 7.9% to 17.5%. Except for *qSNSB1.2* in Bin 149 on chromosome 1, others were detected at both sites and showed a negative effect of the *O. nivara* introgression.

#### Spikelet number per plant

Five QTLs were significantly associated with spikelet number per plant and the phenotypic variation explained by individual QTLs ranged from 6.9% to 13.1%. One QTL, *qSPP1.2*, was detected in Hainan and *O. nivara* introgression showed a positive effect. Two QTLs were detected at both sites, located in Bin 15 on chromosome 1 and Bin 817 on chromosome 9, and both alleles from *O. nivara* reduced spikelet number per panicle.

#### Seed set rate

Six QTLs were identified for the seed set rate. QTL *qSS1*, located in Bin 150 on chromosome 1, was detected in Beijing, explaining 22.6% of the variation in the seed set rate, and the *O. nivara* introgression at this QTL increased the seed set rate. *O. nivara*-derived alleles at the other 5 QTLs showed negative effects in the 93-11 background.

#### Grain length

Five QTLs for GL were detected on chromosomes 1, 3 and 10. The *O. nivara* alleles at *qGL1.1* and *qGL1.2* increased GL and the *O. nivara* alleles at the other 3 QTLs decreased GL. *qGL3.1* was located in Bin 373 on chromosome 3 in Beijing, explaining 17.9% of the phenotypic variation, and showed a negative effect of the *O. nivara* introgression.

#### Grain width

Five QTLs were detected for GW, and the phenotypic variation explained by individual QTLs ranged from 8.0% to 18.5%. The alleles originating from *O. nivara* at all loci reduced GW. The strongest QTL for GW, *qGW2*, was detected in Hainan, with an R^2^ of 18.5%.

#### 1000-grain weight

Six significant QTLs explained 7.5–21.4% of the phenotypic variation, with the *O. nivara* allele increasing grain weight at 2 loci, *qTGW1.2* and *qTGW1.3*, and reducing grain weight at 6 other loci.

#### Yield per plant

Four QTLs for YPP were identified on chromosomes 1, 2 and 4. The *O. nivara* segments at 3 loci increased YPP. The QTL *qYYP1* was detected in Bin 149 on chromosome 1 at Beijing, with a maximum R^2^ of 23.3%.

#### Candidate genes

**Figure 6:**
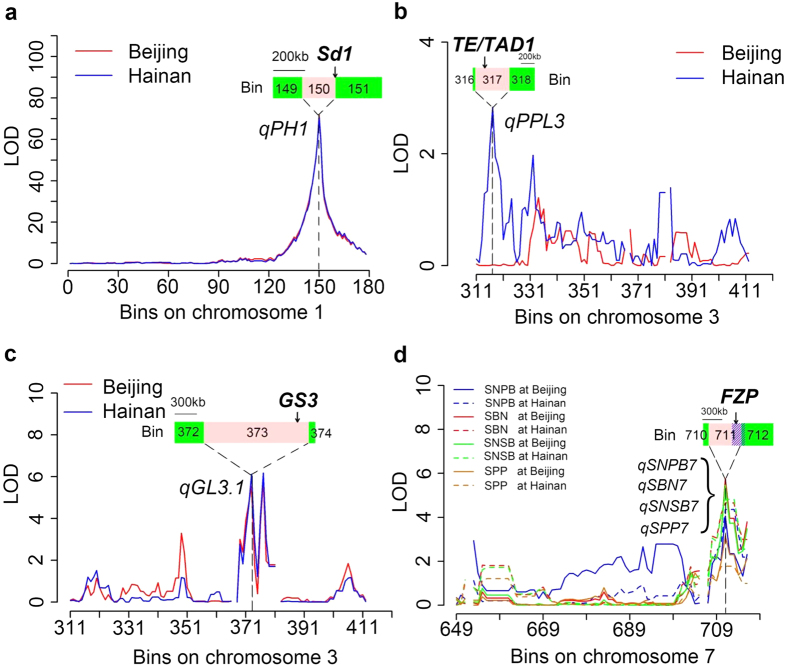
Candidate genes within the peak regions of QTLs for PH (**a**), PPL (**b**), GL (**c**) and a QTL cluster (**d**) for SNPB, SBN, SNSB and SPP. The bin to which an LOD peak corresponds is enlarged along with the adjacent bin and filled with pink. The relative physical locations of candidate genes are indicated by black arrows in the pink bar. Shading lines drawn in the bar indicate selective sweeps overlapping with the bin.

The high-density recombination bin map developed using whole-genome resequencing will greatly improve the accuracy and precision of QTL mapping for the complex traits of rice, as shown in previous studies^24^,^25^,^35^,^36^,^37^. In the present study, the LOD peaks of 7 QTLs were located in small genomic regions containing strong candidate genes cloned previously. The LOD peak of the largest effect QTL, *qPH1*, for PH, covered a region of 200 kb on chromosome 1 that contains the ‘green revolution gene’ *Sd1* (Fig. 6a), which encodes an oxidase enzyme involved in the biosynthesis of gibberellin^38^. The QTL *qPPL3* for PPL was mapped to a small physical interval (1.190–1.698 Mb) on chromosome 3, and a strong candidate gene, *TE/TAD1*, controlling rice tillering by degradation of MONOCULM 1^39^,^40^, is located in this region (Fig. 6b). For GL, the QTL *qGL3.1* was detected in the region from 15.457 to 16.892 Mb that contains the *GS3* gene (Fig. 6c), which controls GL and weight^41^. A QTL cluster on chromosome 7 was associated with SNPB, SBN, SNSB and SPP (Fig. 6d). In the map region of this QTL cluster, there was a strong candidate gene, *FRIZZY PANICLE* (*FZP*), which represses the formation of axillary meristems within the spikelet meristem and promotes the development of floral meristem^42^.

#### Analysis of transgressive ILs

By evaluating these ILs for yield per plant (YPP) at two sites, we identified many transgressive ILs that were superior or inferior to the recipient parent 93-11. Of these ILs, YPP of six ILs outperformed 93-11 by >10% at both sites. Eight ILs in which YPP at both sites was at least 15% lower than that of the recurrent parent 93-11 were identified. QTLs harbored by these transgressive ILs are summarized in Supplementary Table 5. The result showed that the high-yield ILs contained more yield-enhancing QTLs from *O. nivara* than did the low-yield ILs, indicating that the introgression of favorable alleles from *O. nivara* into the elite cultivar had a beneficial effect, increasing grain yield.

## Discussion

*O. nivara* is an annual, photoperiod-insensitive and predominantly self-fertilised wild Asian AA-genome species originally distributed in South and Southeast Asia and constitutes the primary gene pool for improving cultivated rice^43^,^44^. Some valuable genes from *O*. *nivara*, such as those for yield components, grain quality, and grassy stunt virus resistance have been transferred into cultivated rice and widely employed in rice production^20^,^21^,^45^,^46^,^47^. However, owing to the overall inferior performance of *O*. *nivara*, directly identifying and transferring favourable alleles from *O*. *nivara* into elite cultivated rice is challenging^48^. In the present study, we developed a set of ILs derived from a cross between *O*. *nivara* and the elite *indica* variety 93-11. By genotyping each line using whole-genome resequencing, the genomic features of ILs were analysed. The results showed that the 131 ILs covered 94.96% of the *O*. *nivara* genome. Interestingly, many ILs also displayed dramatic phenotypic variation in yield-related traits, compared with the recipient parent 93-11 (Supplementary Figure 5). Therefore, such an introgression library provides an important permanent resource, not only to conserve the genetic resources of *O*. *nivara* in a cultivated rice background but also to evaluate the potential of *O*. *nivara* alleles for improving cultivated rice.

Owing to its characteristics of annual adaptation and seed reproduction, *O. nivara* has more spikelets and larger seed size than the wild perennial Asian AA-genome species *O. rufipogon*. Thus, some agronomically important traits of *O. nivara* are more similar to those of cultivated rice^49^. In a previous study, several significant yield-enhancing QTLs from *O. nivara* were identified by advanced backcross (BC_2_F_2_) QTL analysis^21^. In the present study, by combining a high-density bin map with phenotypic data from ILs, *O*. *nivara* alleles at 24 loci showed positive effects on yield or yield components. For example, the QTL *qPBN8*, associated with PBN, was detected at both sites, explaining 16.1% and 26.6% of the phenotypic variation, respectively. The allele from *O. nivara* at *qPBN8* increased PBN. The *O. nivara*-derived alleles at 3 QTLs for grain YPP showed positive effects in the 93-11 background. These favourable alleles from *O. nivara* have potential use in molecular marker-assisted breeding for improving yield.

During the course of domestication from wild to cultivated rice, many morphological changes have occurred, and desirable changes in agronomic traits have been selected by humans for enhancing rice production, including non-shattering^32^,^33^, erect growth^50^,^51^ and compact panicle architecture^52^,^53^. In an F_2_ population derived from a cross between an *indica* cultivar, CL16, and an *O. nivara* accession, tiller number was controlled by 3 QTLs explaining 5.6–15.0% of the phenotypic variation and *O. nivara*-derived alleles at all 3 loci had positive effects^49^. We detected 5 QTLs for PPL in the present study and found that the *O. nivara* introgression at 4 of the 5 loci increased PPL in the 93-11 background. Tiller number is a key domestication character in rice, and selection results in synchronous tiller production and maturation, increasing productive tiller number and enhancing grain yield. In addition, some QTLs detected in the present study, including *qPH1* for PH, *qPBN2* and *qPBN4* for PBN, *qSBN1.2* and *qSBN7* for SBN, and *qSPP1.2* for SPP, had chromosomal locations similar to those of QTLs previously identified in *O*. *nivara*^21^,^49^,^54^ (Supplementary Table 6). Meanwhile, compared with the reported QTLs detected in the wild perennial species *O. rufipogon*, we found that 15 QTLs for yield-related traits in this study were identified in similar chromosomal regions^9^,^10^,^12^,^15^ (Supplementary Table 6). These results suggested that genetic variation at these QTLs played important roles in the selection of agronomic traits during rice domestication.

Owing to the effect of natural and artificial selection during crop domestication, the diversity of cultivated populations has markedly decreased and selection footprints have been left in the genomes of crop species. Based on the estimates of the ratio of genetic diversity in wild rice to that in cultivated rice, 60, 62 and 55 selection sweeps have been detected between *indica*, *japonica* and cultivated and wild rice, respectively, and 32 QTLs for 15 domestication-associated traits were located within the selection sweeps^55^. By comparing the chromosomal locations of domestication loci with the peak intervals of QTLs, we found that 14 QTLs colocalised with 7 domestication sweeps identified by Huang *et al.*^55^ (Supplementary Table 7). The peak regions of 2 QTLs, *qPBN8* for PBN and *qSNPB8* for SNPB, in the distal region of the long arm of chromosome 8 were colocalised within the domestication sweep (23.7–23.8 Mb) with a strong selection signal 13.9 (the genome-wide threshold of a selection signal is a π_w_/π_c_ value greater than 3). These results implied that comparison of the locations of selective chromosomal regions with those of QTLs would further annotate the domestication features of selection sweeps. In future, molecular cloning and evolutionary analyses of domestication-associated QTLs will shed light on rice domestication.

## Methods

### Plant materials

The recipient parent 93-11 is the *indica* rice variety with genome sequence^56^ and widely grown in China. The donor parent W2014 is an annual wild rice accession (*O. nivara*) collected from India (20°18′N, 72°55′E) and maintained at the National Institute of Genetics, Japan. To construct the IL population, we selected single plant from the recipient parent 93-11 and the donor parent *O. nivara* (W2014), respectively, for crossing, and obtained 23 F_1_ plants. All F_1_ plants were backcrossed three times in succession to 93-11 to yield a BC_3_F_1_ population of 256 individuals. Based on the genotypes of BC_3_F_1_ characterized with 120 polymorphic SSR markers from across the whole genome (data not shown), 150 BC_3_F_1_ were selected and self-pollinated for six generations to yield an IL population of 131 lines. The development of the ILs is illustrated in Supplementary Figure 6. The ILs and parents are available via material transfer agreement for research purposes or via license for commercial purposes.

### Identification of single-nucleotide polymorphism (SNPs) and construction of a bin map

Genomic DNA of both parents and the 131 ILs was used to construct a DNA library that was sequenced on an Illumina HiSeq2500 platform. All reads were mapped to the reference genome (Os-Nipponbare-Reference-IRGSP-1.0, MSU release 7) with Burrows-Wheeler Aligner (BWA) tools, which efficiently align low-divergent sequences against a large reference genome^57^. SNPs between the two parents were identified with SAMtools^30^ using the following criteria: i). SNPs must be identified in uniquely mapped reads; ii) the base quality score must exceed 25, and iii) the mapping alignment qualities of the parents and IL must exceed 40 and 20, respectively. To construct a high-quality bin map, only SNPs that met the following criterion were retained: i) the distance between adjacent SNPs was at least 10 bp, ii) each SNP allele was covered by at least 4 reads, and iii) only biallelic SNPs were used. The variant effects of these high quality SNPs were predicted with SnpEff^31^.

The missing genotypes of each IL were imputed with the *k*-nearest neighbor algorithm^58^. A slightly modified sliding-window analysis with a 17-SNP window size and 1-SNP increment was used to determine individual genotypes and breakpoints located at genotype transitions^24^, as follows: a window with a 93-11:W2014 allele ratio of 14:3 or higher was defined as having a homozygous 93-11 genotype, one with a ratio of 5:12 or lower was defined as having a homozygous W2014 genotype, and others with intermediate ratios were defined as heterozygous. Consecutive windows with the same genotype were then combined into blocks and a recombination breakpoint was assumed at the transition between two different genotype blocks. According to individual genotype and recombination breakpoint information, a skeleton bin map was generated for QTL analysis.

### Phenotypic evaluation

The population was planted in experimental fields in Beijing (39°54′N, 116°28′E) in the summer season of 2013 and in Sanya (18°14′N, 109°31′E), Hainan province, China, in the autumn of 2013. Each IL was planted in 4 rows at 10 plants per row. Ten plants were randomly selected from the middle two rows to be evaluated for 13 yield-associated and 2 domestication associated traits, including plant height (PH), panicles per plant (PPL), panicle length (PL), primary branch number (PBN), spikelet number on primary branch (SNPB), secondary branch number (SBN), spikelet number on secondary branch (SNSB), spikelets per panicle (SPP), seed set (SS), grain length (GL), grain width (GW), 1000-grain weight (TGW), yield per plant (YPP), seed shattering (SH) and hull colour (HC). The evaluation methods for each trait are briefly described in Supplementary Table 8. Among the traits, PL, PBN, SNPB, SBN, SNSB and SPP were measured in the main panicle.

### Data analysis

The R function cor using the Pearson method was used to calculate trait correlations, and significance testing was performed with the corr.test function from the psych package (R Development Core Team, 2013).

Using bins as single markers, QTL analysis was performed with QTL IciMapping V4.0^59^. The LOD threshold was set as 2.0. All QTLs were named according to the nomenclature described by McCouch *et al.*^60^.

The domestication-associated chromosomal regions identified by Huang *et al.*^55^ were anchored to the reference genome Nipponbare (Os-Nipponbare-Reference-IRGSP-1.0, MSU release 7) by BLAST analysis. Comparison and overlap analysis between the domestication loci and peak intervals of QTL was performed with a custom Python script.

## Additional Information

**How to cite this article**: Ma, X. *et al.* Genomic structure analysis of a set of *Oryza nivara* introgression lines and identification of yield-associated QTLs using whole-genome resequencing. *Sci. Rep.* **6**, 27425; doi: 10.1038/srep27425 (2016).

## Supplementary Material

Supplementary Information

Supplementary Dataset 1

Supplementary Dataset 2

Supplementary Dataset 3

## Figures and Tables

**Table 1:** QTLs identified for 13 yield-related traits using the high density bin map.

Trait	QTL	Chrom.	Bin^a^	Beijing			Hainan		
				LOD	*R*^*2*^ (%)^b^	Additive^c^	LOD	*R*^*2*^ (%)	Additive
PH	*qPH1*	1	Bin150	71.9	92.0	45.9	70.9	91.9	39.2
	*qPH3*	3	Bin407	2.6	8.8	21.6	2.8	9.3	19.0
PPL	*qPPL1.1*	1	Bin40				2.0	6.9	0.7
	*qPPL1.2*	1	Bin150				10.0	29.9	−0.8
	*qPPL2*	2	Bin298				2.6	8.7	0.6
	*qPPL3*	3	Bin317				2.8	9.6	0.8
	*qPPL10*	10	Bin889				2.5	8.3	0.6
PL	*qPL1*	1	Bin150	26.6	60.7	2.5	10.6	31.4	1.2
	*qPL3*	3	Bin329				3.8	12.6	−1.7
	*qPL4*	4	Bin456	2.4	8.2	−1.4	2.6	8.9	−0.9
	*qPL8*	8	Bin788	2.5	8.4	1.6	2.4	8.1	1.0
	*qPL11*	11	Bin969	3.1	10.2	2.4			
	*qPL12*	12	Bin1059				2.9	9.6	1.1
PBN	*qPBN1*	1	Bin99	4.0	13.0	−0.6			
	*qPBN2*	2	Bin245	7.7	23.7	−0.9			
	*qPBN4*	4	Bin462	2.9	9.6	−0.6	2.5	8.3	−0.5
	*qPBN8*	8	Bin781	5.0	16.1	0.7	8.7	26.6	0.9
	*qPBN11*	11	Bin950				2.3	7.8	1.1
SNPB	*qSNPB1*	1	Bin29				2.3	7.8	5.9
	*qSNPB2*	2	Bin252	4.2	13.6	−5.1			
	*qSNPB4.1*	4	Bin463	4.0	13.0	−6.5			
	*qSNPB4.2*	4	Bin470				3.7	12.1	−7.3
	*qSNPB6*	6	Bin647				2.1	7.2	−4.5
	*qSNPB7*	7	Bin711	4.1	13.3	7.5	4.4	14.3	7.3
	*qSNPB8*	8	Bin781	7.5	23.1	7.4	9.3	28.1	7.7
SBN	*qSBN1.1*	1	Bin15	2.8	9.3	−4.5	2.2	7.6	−3.0
	*qSBN1.2*	1	Bin149				3.6	12.1	2.4
	*qSBN2*	2	Bin224	2.5	8.5	−5.2			
	*qSBN7*	7	Bin711	5.8	18.4	−7.7	4.7	15.2	−5.2
	*qSBN9*	9	Bin817	3.8	12.5	−4.3	3.1	10.5	−2.9
SNSB	*qSNSB1.1*	1	Bin15	2.9	9.7	−19.1	2.3	7.9	−12.1
	*qSNSB1.2*	1	Bin149				2.5	8.4	7.9
	*qSNSB7*	7	Bin711	5.5	17.5	−31.1	4.8	15.7	−20.7
	*qSNSB9*	9	Bin817	4.8	15.5	−19.8	3.7	12.4	−12.5
SPP	*qSPP1.1*	1	Bin15	3.2	10.6	−19.6	2.0	6.9	−11.8
	*qSPP1.2*	1	Bin149				3.0	10.2	9.1
	*qSPP2*	2	Bin213	2.5	8.3	−18.3			
	*qSPP7*	7	Bin711	3.2	10.5	−23.6			
	*qSPP9*	9	Bin817	4.0	13.1	−17.8	2.1	7.0	−9.8
SS	*qSS1*	1	Bin150	6.8	22.6	0.1			
	*qSS2*	2	Bin298	2.7	9.8	−0.1			
	*qSS3*	3	Bin332	2.7	9.6	−0.1	2.5	8.9	−0.04
	*qSS4*	4	Bin492				6.5	22.0	−0.1
	*qSS6*	6	Bin635				3.4	12.1	−0.1
	*qSS8*	8	Bin793	2.6	9.4	−0.04			
GL	*qGL1.1*	1	Bin111				3.5	11.8	0.3
	*qGL1.2*	1	Bin145	4.2	13.9	0.2			
	*qGL3.1*	3	Bin373	5.5	17.9	−0.7	6.1	19.9	−0.6
	*qGL3.2*	3	Bin377	5.9	18.9	−0.7	6.2	20.1	−0.6
	*qGL10*	10	Bin896	2.8	9.5	−0.3			
GW	*qGW2*	2	Bin284				5.6	18.5	−0.04
	*qGW8*	8	Bin794				4.5	14.9	−0.1
	*qGW11.1*	11	Bin980				2.9	10.0	−0.04
	*qGW11.2*	11	Bin983	2.3	8.0	−0.1			
	*qGW12*	12	Bin1025				3.2	11.0	−0.03
TGW	*qTGW1.1*	1	Bin38	4.1	16.2	−2.3	2.1	7.5	−1.2
	*qTGW1.2*	1	Bin148	3.4	13.6	1.1	3.2	11.0	0.9
	*qTGW1.3*	1	Bin162				3.8	13.0	1.0
	*qTGW2*	2	Bin310	2.2	9.0	−1.7	5.1	17.2	−1.8
	*qTGW3*	3	Bin377	2.3	9.7	−2.3	6.5	21.4	−3.0
	*qTGW10*	10	Bin895	5.3	20.5	−1.8	3.5	12.1	−1.1
YYP	*qYYP1*	1	Bin149	6.2	23.3	3.5			
	*qYYP2*	2	Bin182	2.1	8.7	4.1			
	*qYYP4.1*	4	Bin494				2.1	7.7	−3.4
	*qYYP4.2*	4	Bin505				3.1	11.1	7.0

^a^Bin overlapping with the LOD peak of the QTL.

^b^Percentage of phenotypic variation explained by individual QTL.

^c^Additive effect of each QTL from *O. nivara*.
