# Supplementary material for: Genomic structure analysis of a set of Oryza nivara introgression lines and identification of yield-associated QTLs using whole-genome resequencing
Source: Sci Rep. 2016 Jun 2;6:27425. doi: 10.1038/srep27425 (PMC4890301; doi:10.1038/srep27425)
Supplement: Supplementary Information [file srep27425-s1.pdf]

Genomic structure analysis of a set of *Oryza nivara* introgression lines and identification of yield-associated QTLs using whole-genome resequencing

Xin Ma[1], Yongcai Fu[1], Xinhui Zhao[1, 2], Liyun Jiang[1], Zuofeng Zhu[1], Ping Gu[1], Wenying Xu[2], Zhen Su[2], Chuanqing Sun[1, 2] and Lubin Tan[1*]

[1]National Center for Evaluation of Agricultural Wild Plants (Rice), Beijing Key Laboratory of Crop Genetic Improvement, Laboratory of Crop Heterosis and Utilization, MOE, Department of Plant Genetics and Breeding, China Agricultural University, Beijing 100193, China.

[2]State Key Laboratory of Plant Physiology and Biochemistry, China Agricultural University, Beijing 100193, China.

*Correspondence and requests for materials should be addressed to L.T. (email: tlb9@cau.edu.cn)

Supplementary information

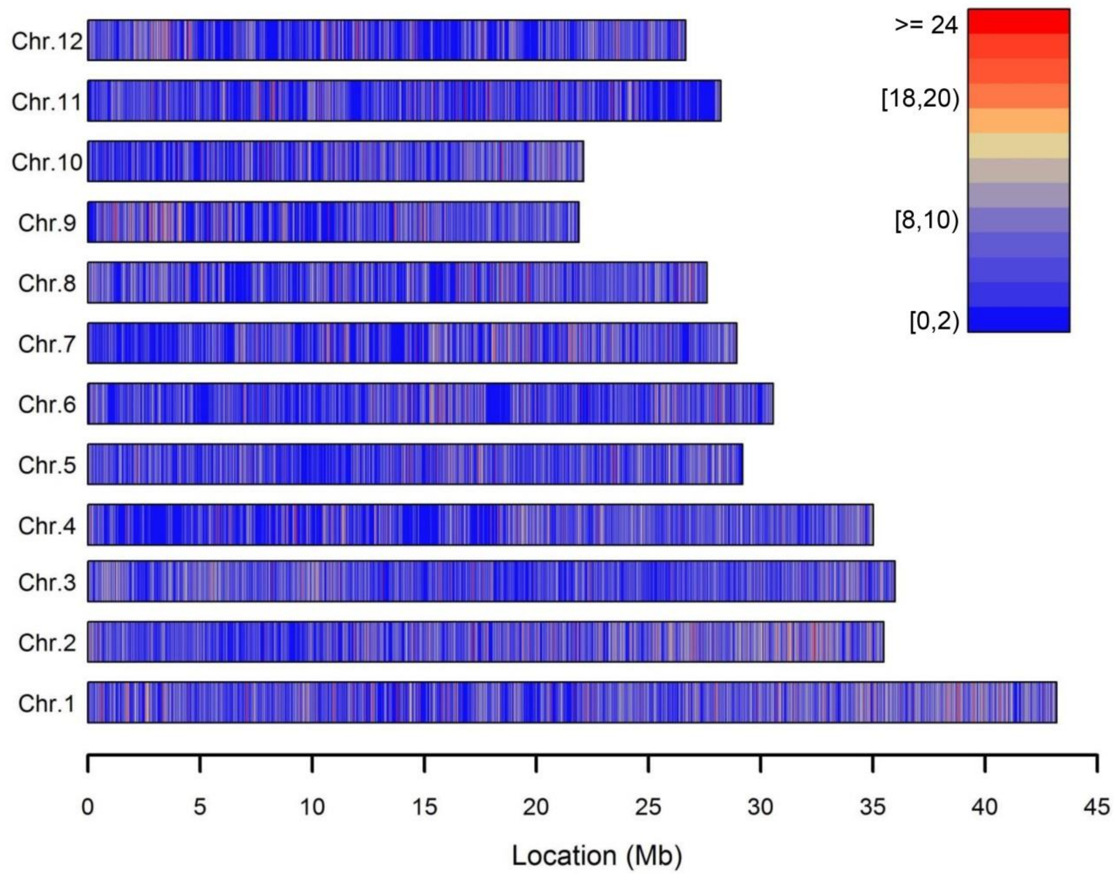

Supplementary Figure 1. Distribution of high quality SNPs along 12 chromosomes. The colour from blue to red represents the number of SNPs in each 10kb physical interval.

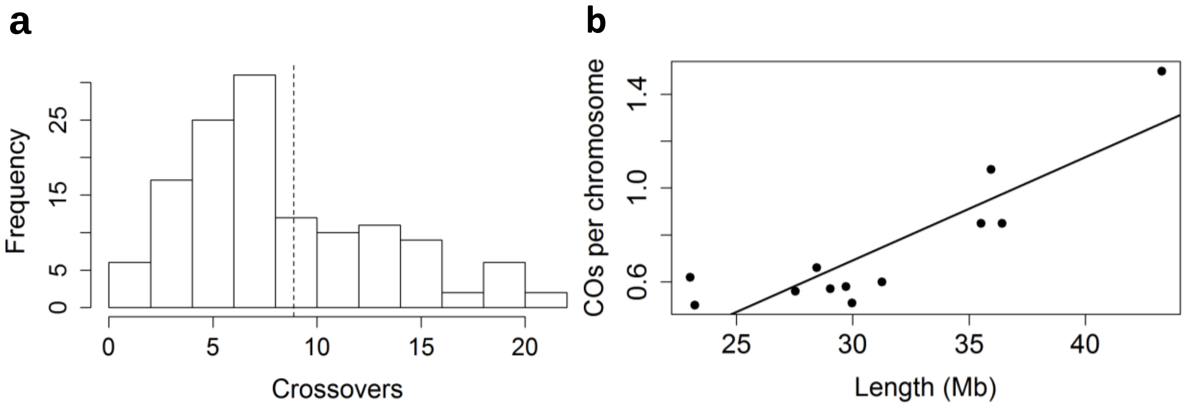

Supplementary Figure 2. COs identified in ILs. (a) A total of 1164 COs detected with an average of 8.89 (black dashed line indicates the mean value). (b) Correlation between the average numbers of COs per chromosome and physical size of a chromosome: $Y = 0.044X - 0.6315$, $R^2 = 0.738$.

a

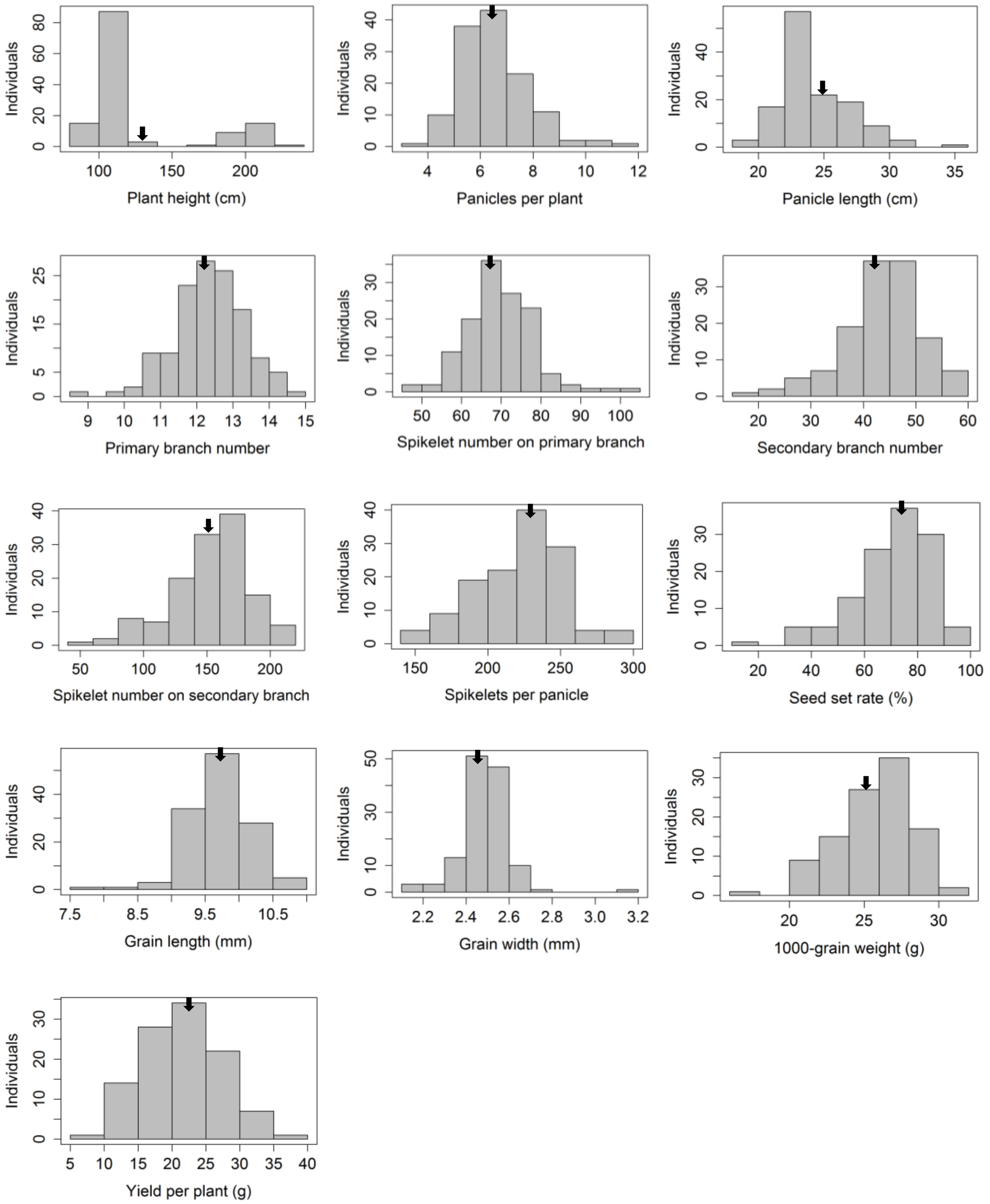

Supplementary Figure 3. Frequency distribution of 13 yield-associated traits in Beijing (a) and Hainan (b). Population means are indicated by black arrows.

Supplementary Figure 3. Continued

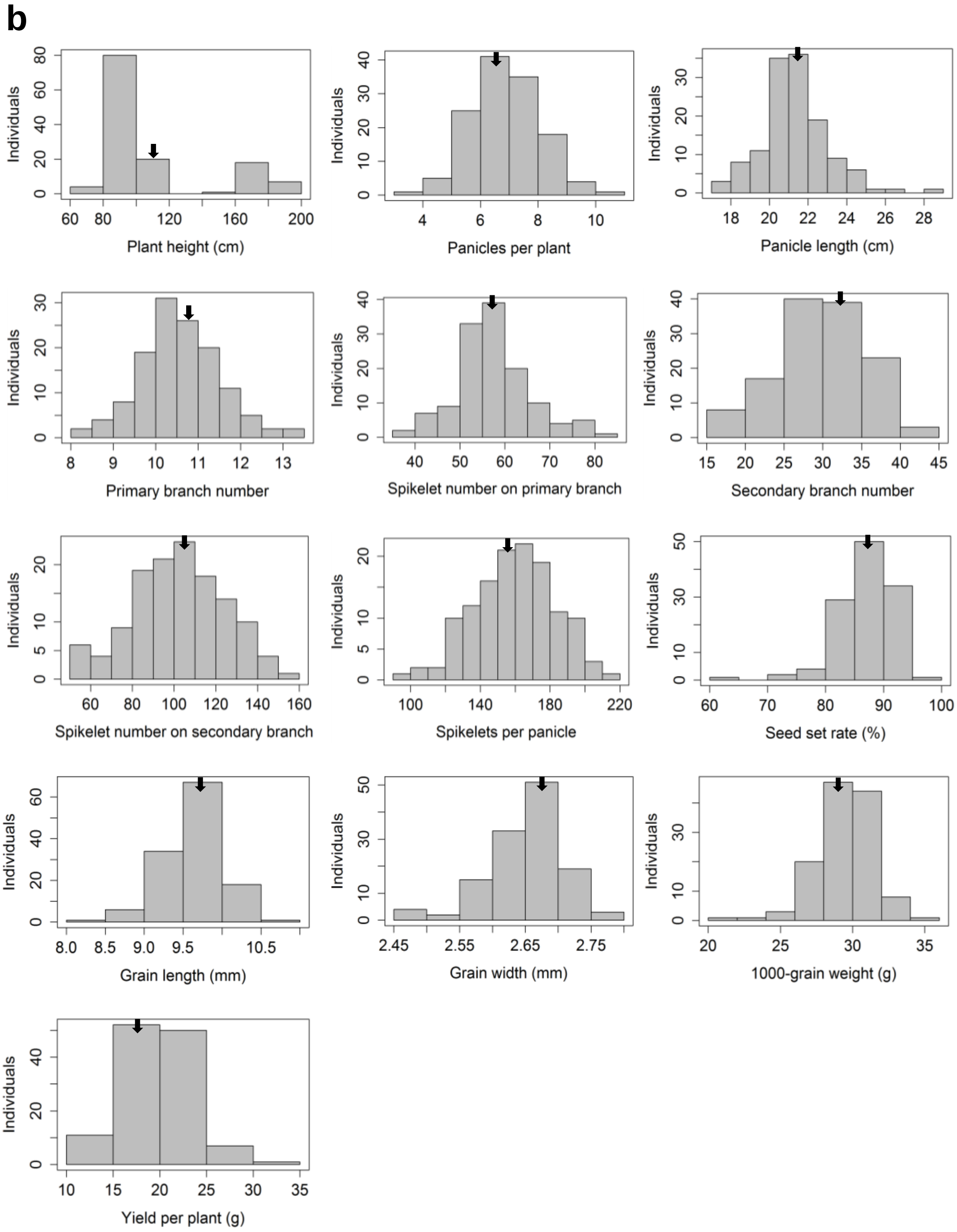

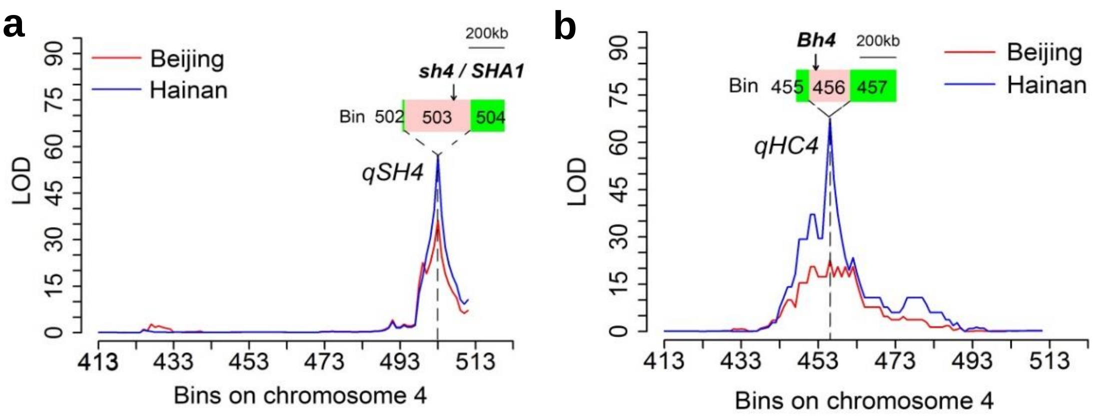

Supplementary Figure 4. Locations of major QTLs for seed shattering (a) and hull colour (b) with strong candidate gene at Beijing (red line) and Hainan (blue line). The bin to which an LOD peak corresponds is enlarged along with the adjacent bin and filled with pink. The relative physical locations of strong candidate genes are indicated by black arrows in the pink bin.

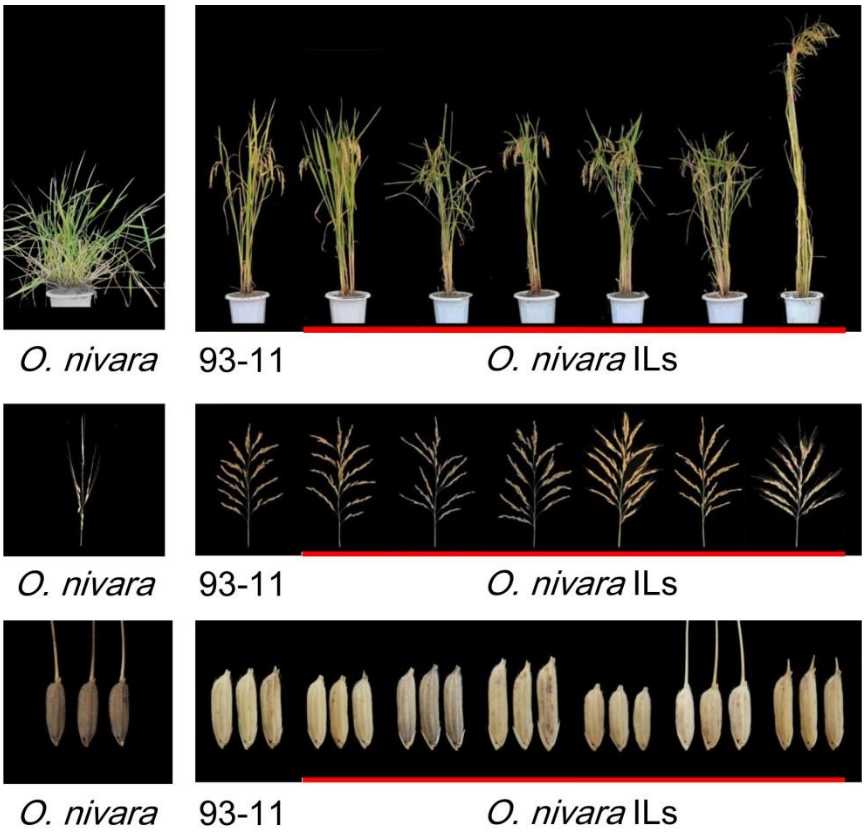

Supplementary Figure 5. Phenotypic variation in plant height, panicle architecture, and seed size among *O. nivara*, 93-11, and introgression lines.

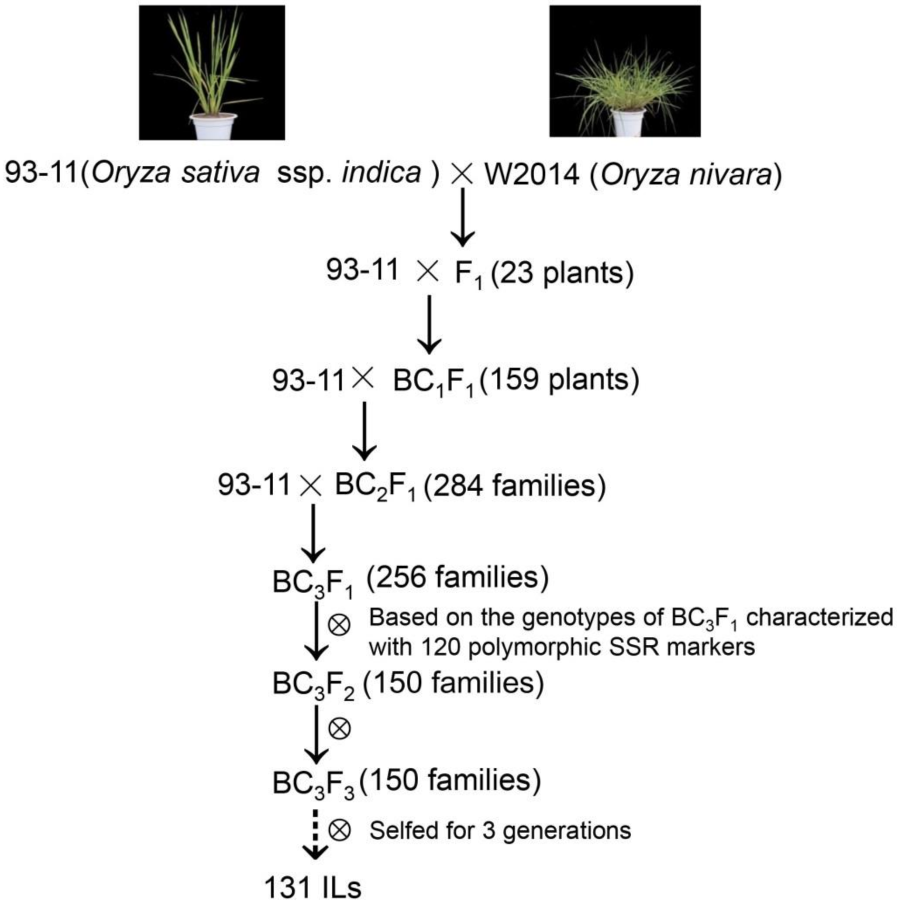

Supplementary Figure 6. Flow chart of the development of *O. nivara* ILs.

Supplementary Table 1. Distribution of high-quality SNPs on 12 chromosomes.

Chrom.	SNP distribution						Genes	
	Total	Promoter[a]	Gene	CDS	5′-UTR	3′-UTR	Numbers	Mutated
1	25551	6118	13487	4265	345	1490	6529	3084
2	20936	5043	10641	3434	267	1193	5377	2593
3	18581	4432	9732	2944	192	1094	5568	2748
4	15276	3476	7811	2756	242	730	5317	2012
5	13838	3178	6662	2360	155	727	4572	1958
6	13679	3201	7001	2529	120	606	4709	1794
7	13241	2817	6921	2522	120	663	4450	1738
8	14371	3195	6973	2478	152	633	4188	1672
9	11744	2686	5590	2072	111	482	3407	1348
10	11121	2494	5433	2111	114	494	3510	1297
11	11441	2560	5812	2458	126	417	4160	1431
12	12178	2526	6064	2291	137	550	4014	1343
Total	181957	41726	92127	32220	2081	9079	55801	23018

[a] upstream 2-kb region of the gene

Supplementary Table 2. Statistical analysis of *O. nivara* introgressed segments in ILs and proportion of donor genome represented by homozygous and heterozygous segments.

Chrom.	Heterozygous segments		Homozygous segments		Percentage of Homozygous Segments	Maximum percentage genome coverage (Het + Homo)
	Number of segments	Average segment length (Mb)	Number of segments	Average segment length (Mb)		
1	15	1.63	102	4.97	92.32	100
2	16	0.86	69	4.23	81.33	92
3	23	1.69	48	2.27	47.61	85.4
4	22	0.94	56	3.48	92	100
5	19	1.53	27	2.88	71.23	92.27
6	21	1.08	37	2.07	62.04	88.07
7	17	0.96	34	5.39	73.46	90.23
8	5	0.54	49	5.36	100	100
9	13	1.25	50	5.72	93.89	100
10	1	0.93	38	6.33	96.01	100
11	22	1.15	35	2.50	72.42	94.28
12	0	0	48	6.36	100	100
Average	1.33	1.14	4.5	4.3	81.07	94.96

Supplementary Table 3. Correlation coefficients among 13 yield-associated traits at the site in Beijing.

	PH	PPL	PL	PBN	SNPB	SBN	SNSB	SPP	SS	GL	GW	TGW
PPL	−0.13											
PL	0.83**	−0.19										
PBN	0.06	−0.06	0.11									
SNPB	0.17	−0.11	0.34**	0.69**								
SBN	0.23**	0.03	0.21*	0.37**	−0.15							
SNSB	0.27**	0.04	0.25**	0.26**	−0.21*	0.97**						
SPP	0.33**	0.01	0.35**	0.46**	0.07	0.95**	0.96**					
SS	0.52**	−0.27**	0.50**	0.00	0.03	0.19*	0.17	0.19*				
GL	0.39**	−0.10	0.44**	−0.10	0.13	−0.11	−0.08	−0.04	0.22*			
GW	−0.17	−0.08	−0.18*	−0.10	0.02	−0.12	−0.12	−0.12	−0.09	−0.20*		
TGW	0.42**	−0.30**	0.36**	−0.10	−0.02	0.06	0.06	0.06	0.64**	0.49**	0.11	
YPP	0.50**	0.37**	0.42**	0.11	−0.06	0.52**	0.55**	0.54**	0.66**	0.11	−0.12	0.44**

* Significant at $P < 0.05$.

** Significant at $P < 0.01$.

Supplementary Table 4. Correlation coefficients among 13 yield-associated traits at the site in Hainan.

	PH	PPL	PL	PBN	SNPB	SBN	SNSB	SPP	SS	GL	GW	TGW
PPL	−0.56**											
PL	0.63**	−0.42**										
PBN	0.30**	−0.23**	0.45**									
SNPB	0.12	−0.12	0.45**	0.75**								
SBN	0.40**	−0.45**	0.36**	0.45**	0.00							
SNSB	0.33**	−0.40**	0.32**	0.36**	−0.07	0.97**						
SPP	0.36**	−0.42**	0.47**	0.62**	0.28**	0.94**	0.94**					
SS	0.18*	−0.16	0.11	−0.09	−0.11	0.09	0.07	0.02				
GL	0.32**	−0.27**	0.27**	0.01	0.11	−0.15	−0.20*	−0.15	0.01			
GW	−0.03	−0.08	−0.23**	−0.18*	−0.08	−0.11	−0.14	−0.16	0.14	−0.14		
TGW	0.37**	−0.34**	0.27**	−0.01	0.12	−0.05	−0.11	−0.06	0.11	0.61**	0.41**	
YPP	0.00	0.27**	0.19*	0.18*	0.01	0.38**	0.38**	0.36**	0.15	−0.03	−0.02	0.13

* Significant at $P < 0.05$.

** Significant at $P < 0.01$.

Supplementary Table 5. Transgressive ILs and QTLs included in the introgressed segments.

ILs	Percentage over recipient parent (%)[a]		QTLs detected	
	Beijing	Hainan	Positive QTLs	Negative QTLs
**High Yield**				
IL30	21.0	47.6	*qPH1*, *qPL1*, *qSBN1.2*, *qSNSB1.2*, *qSPP1.2*, *qSS1*, *qGL1.1*, *qGL1.2*, *qTGW1.2*, *qTGW1.3*, *qYYP1*, *qYYP2*	*qPPL1.2*
IL58	14.0	15.2	*qPH1*, *qPL1*, *qPL12*, *qSBN1.2*, *qSNSB1.2*, *qSPP1.2*, *qSS1*, *qGL1.1*, *qGL1.2*, *qTGW1.2*, *qYYP1*	*qPPL1.2*, *qSBN9*, *qSNSB9*, *qSPP9*
IL82	25.3	21.3		
IL91	12.5	15.9		
IL112	18.0	12.1		
IL124	48.4	20.5	*qPH1*, *qPL1*, *qSBN1.2*, *qSNSB1.2*, *qSPP1.2*, *qSS1*, *qGL1.2*, *qTGW1.2*, *qTGW1.3*, *qYYP1*, *qPL8*, *qPBN8*, *qSNPB8*	*qPPL1.2*, *qGL10*, *qTGW10*
**Low Yield**				
IL28	-20.6	-25.2	*qTGW1.3*	*qPL4*, *qPBN4*, *qSNPB4.1*, *qSNPB4*, *qYYP4.1*
IL33	-16.9	-22.5		*qSNPB6*, *qSBN1.1*, *qSNSB1.1*, *qSPP1.1*
IL40	-23.9	-21.0	*qPPL10*	*qGL10*, *qTGW10*
IL41	-18.0	-15.2		
IL53	-18.0	-29.8		
IL55	-17.9	-17.4	*qPBN8*, *qPBN11*, *qSNPB8*	*qGW12*
IL64	-27.6	-16.2	*qPL8*, *qPL11*, *qPBN8*, *qSNPB8*	*qSS8*, *qGW8*, *qGW11.1*
IL75	-52.9	-39.3		*qPL4*, *qPBN4*, *qSNPB4.1*, *qGW11.2*

[a] Percentage over recipient parent = 100*(IL – 93-11) / 93-11

Supplementary Table 6. Comparison of QTLs controlling yield-related traits in this study with those reported in previous studies.

			QTLs identified in previous studies						
Chrom.	Maker interval	QTLs identified in this study[a]	Li *et al*. (2006)[49]	Swamy *et al*. (2014)[21, b]	Eizenga *et al*. (2016)[54]	Septiningsih *et al*. (2003)[10]	Thomson *et al*. (2003)[9]	Tan *et al*. (2007)[15]	Wickneswari *et al*. (2012)[12]
1	Bin15-Bin29	*qSBN1.1*, *qSNSB1.1*, *qSNPB1*, ***qSPP1.1***.	*tn1*	*gw1.1*(2)		*gpl1.1*, *yld1.1*	***spp1.1***, *gpp1.1*	*ph1.1*	***qSPP-1***, *qGPP-1*, *qYLD-1*, *qSPL-1*
	Bin38-Bin40	*qPPL1.1*, *qTGW1.1*				*yld1.2*	*ph1.1*	*gpp1.1*, *pss1.1*	
	Bin99-Bin111	*qPBN1*, *qGL1.1*							
	Bin145-Bin162	***qPH1***, ***qPPL1.2***, ***qPL1***, ***qSBN1.2***, *qSNSB1.2*, ***qSPP1.2***, ***qSS1***, *qGL1.2*, ***qTGW1.2,*** ***qTGW1.3***, ***qYYP1***	*pbr1*, ***sbr1***, ***sn1***.	***ph1.1***, ***ntp1.1***(2), ***nsp1.1***, *nfg1.1*.		***ppl1.1***, ***pss1.1***, ***ph1.1***, ***gw1.1***, ***ppl1.2***, ***pl1.1***.	***ph1.2***, ***pss1.1***, ***gw1.1***	***ppl1.1***, ***gpp1.2***, ***gyp1.1***, ***ph1.2***, ***kgw1***.	*qCL-1-2*, ***qPL-1***, ***qPH-1-2***
2	Bin182	*qYYP2*						*spp2.1*, *gpp2.1*	
	Bin213-Bin224	*qSBN2*, ***qSPP2***		*gw2.2*(1)			*gpp2.1*, ***spp2.1***, *yld2.1*		
	Bin245-Bin252	***qPBN2***, *qSNPB2*	***pbr2***	*nt2.1*(1)					
	Bin284-Bin310	***qPPL2***, ***qSS2,*** *qGW2*, ***qTGW2***		*yldp2.1*(2), *nsp2.1*(1), *nfg2.1(1)*, *yldp2.1*(1), *ph2.1*(1)		***pss2.1***, *gpp2.1*, *yld2.1*, *gw2.1*	***gw2.1***, *pl2.1*	*spp2.2*, ***kgw2.1***	*qTPL-2*, ***qPPL-2***, *qSPP-2*, *qGPP-2*.
3	Bin317-Bin332	*qPPL3*, *qPL3*, *qSS3*		*gw3.1*(1)					*qCL-3*, *qPH-3*, *qSPL-3* *qGW-3-1*, *qSPP-3*
	Bin373-Bin377	*qGL3.1*, *qGL3.2*, ***qTGW3***					*yld3.1*, ***gw3.1***		***qGW-3-2***
	Bin407	*qPH3*				*gw3.2*	*spp3.1*, *gpp3.1*, *yld3.2*, *pss3.1*, *gw3.2*	*gpp3.1*, *ppl3.1*	
4	Bin456	*qPL4*							

Supplementary Table 6. Continued

			QTLs identified in previous studies						
Chrom.	Maker interval	QTLs identified in this study[a]	Li *et al.* (2006)[49]	Swamy *et al.* (2014)[21, b]	Eizenga *et al.* (2016)[54]	Septiningsih *et al.* (2003)[10]	Thomson *et al.* (2003)[9]	Tan *et al.* (2007)[15]	Wickneswari *et al.* (2012)[12]
	Bin462-Bin470	***qPBN4***, *qSNPB4.1*, *qSNPB4.2*	***pbr4***, *sbr4*, *sn4*, *pl4*, *ph4*.		*qSPP4*, *qSS4*.				
	Bin492-Bin505	***qSS4***, *qYYP4.1*, *qYYP4.2*		*gw4.2*(1)			*gpp4.1*, ***pss4.1***	*ppl4.1*, *spp4.1*	
6	Bin635-Bin647	*qSNPB6*, *qSS6*		*npt6.1*(1)					
7	Bin711	*qSNPB7*, ***qSBN7***, *qSNSB7*, *qSPP7*	***sbr7.2***						
8	Bin781	*qPBN8*, *qSNPB8*					*gpp8.1*, *ph8.1*		
	Bin788-Bin794	*qPL8*, *qSS8*, *qGW8*							
9	Bin817	*qSBN9*, *qSNSB9*, *qSPP9*						*kgw9.1*	
10	Bin889-Bin896	*qPPL10*, *qTGW10*, *qGL10*							
11	Bin950	*qPBN11*				*ppl11.1*			
	Bin969-Bin983	*qPL11*, *qGW11.1*, *qGW11.2*		*nt11.1*(1), *yldp11.1*(1), *yldp11.1*(2)		*ppl11.2*		*kgw11.1*	
12	Bin1025	*qGW12*					*gpp12.1*	*ph12.1*, *ppl12.1* *spp12.1* , *gpp12.1*	
	Bin1059	*qPL12*							

[a] QTLs in bold detected in this study shared similar regions with those reported in previous studies.

[b] The number (1 and 2) inside parentheses represents that the QTL was detected using Population 1 and Population 2, respectively, and other QTLs identified using both populations by Swamy *et al*. (2014).

Supplementary Table 7. QTLs colocalised with previously reported domestication-associated chromosomal regions.

Chrom.	Previous study (Huang *et al.*[55])		The present study
	π_w/π_c [a]	Physical interval[b] (Mb)	QTL[c]
2	5.2	17.6-17.7	*qHC2*
3	3.7	34.9-35.0	*qPH3*
4	6.4	22.9-23.4	*qPL4; qHC4*
4	9.4	25.4-27.1	*qPBN4;qSNPB4.1;qSNPB4.2*
4	3.6	33.9-34.3	*qSH4*
7	4.8	28.1-28.3	*qSNPB7;qSBN7;qSNSB7;qSPP7*
8	13.9	23.7-23.8	*qPBN8;qSNPB8*

[a] π_w/π_c value was estimated by the ratio of genetic diversity in wild rice to that in cultivated rice.

[b] Physical interval is anchored to the reference genome Nipponbare (Os-Nipponbare-Reference-IRGSP-1.0, MSU release 7) by BLAST analysis of the sequence from the selective sweep regions from previous study.

[c] QTLs overlapping with the selective sweep region.

Supplementary Table 8. Description of the qualitative and quantitative traits surveyed.

Trait	Abbreviation	Evaluation method
**Qualitative traits**		
Seed shattering	SH	Shattering or nonshattering
Hull colour	HC	Black or yellow hull
**Quantitative traits**		
Plant height	PH	The average height of 10 plants from the field surface to the top of the plants at ripening age (excluding the awn)
Panicles per plant	PPL	The average tiller number of 10 plants at the time of harvest (panicles having fewer than 5 seeds were not counted)
Panicle length	PL	The average length from the panicle neck to the panicle tip (excluding the awn) on the main panicles of 10 plants
Primary branch number	PBN	The average number of primary branches on the main panicles of 10 plants
Spikelet number on primary branch	SNPB	The average number of spikelets on the primary branches of the main panicles of 10 plants
Secondary branch number	SBN	The average number of secondary branches on the main panicles of 10 plants
Spikelet number on secondary branch	SNSB	The average number of spikelets on the secondary branches of the main panicles of 10 plants
Spikelets per panicle	SPP	The average number of spikelets on the main panicles of 10 plants
Seed set rate	SS	The number of filled grains divided by the total spikelet number per main panicle evaluated for the main panicle on each of the 10 plants
Grain length	GL	The length from the grain base to the tip (excluding the awn)
Grain width	GW	The width of the widest part of the grain
1000-grain weight	TGW	The average weight of 1000 filled grains per plant calculated for 10 plants
Yield per plant	YPP	The average weight of the bulk harvested grain from 10 plants
